# Perceived discrimination and health outcomes among Asian Indians in the United States

**DOI:** 10.1186/s12913-016-1821-8

**Published:** 2016-10-12

**Authors:** Ranjita Misra, Haslyn Hunte

**Affiliations:** Department of Social and Behavioral Sciences, School of Public Health, West Virginia Univeristy, 1 Medical Center Drive, PO Box 9190, Morgantown, WV 26506-9190 USA

**Keywords:** Discrimination, Asian Indian, Health status, Race & ethnicity, National surveys

## Abstract

**Background:**

Perceived interpersonal discrimination while seeking healthcare services is associated with poor physical and mental health. Yet, there is a paucity of research among Asian Americans or its subgroups.

This study examined the correlates of reported interpersonal discrimination when seeking health care among a large sample of Asian Indians, the 3rd largest Asian American subgroup in the US, and identify predictors of adverse self-rated physical health, a well-accepted measure of overall health status.

**Methods:**

Cross-sectional survey. Participants comprised of 1824 Asian Indian adults in six states with higher concentration of Asian Indians.

**Results:**

Mean age and years lived in the US was 45.7 ± 12.8 and 16.6 ± 11.1 years respectively. The majority of the respondents was male, immigrants, college graduates, and had access to care. Perceived interpersonal discrimination when seeking health care was reported by a relatively small proportion of the population (7.2 %). However, Asian Indians who reported poor self-rated health were approximately twice as likely to perceived discrimination when seeking care as compared to those in good or excellent health status (OR 1.88; 95 % CI 1.12–3.14). Poor self-rated health was associated with perceived health care discrimination after controlling for all of the respondent characteristics (OR 1.93; 95 % CI: 1.17–3.19). In addition, Asian Indians who lived for more than 10 years in the U.S. (OR 3.28; 95 % CI: 1.73–6.22) and had chronic illnesses (OR 1.39; 95 % CI: 1.17–1.64) (*p* < 0.05) were more likely to perceive discrimination when seeking health care. However, older Asian Indians, over the age of 55 years, were less likely to perceive discrimination than those aged 18–34 years Indian American.

**Conclusion:**

Results offers initial support for the hypothesis that Asian Indians experience interpersonal discrimination when seeking health care services and that these experiences may be related to poor self-rated health status.

## Background

Perceived interpersonal discrimination, a hypothesized psychosocial stressor based on the perception on poor or unfair treatment when compared to others, is strongly associated with poor overall physical and mental health among racial/ethnic minority groups [1], and Whites [2]. Evidence from recent literature reviews [3, 4] and meta-analysis [5] suggest that perceived interpersonal discrimination is associated with a myriad of health behaviors and outcomes among various of racial/ethnic minority groups and even among select groups of Whites [6]. Specifically, health outcomes associated with perceived interpersonal discrimination have varied widely from alcohol/tobacco use, [7–11] hypertension/blood pressure, [12–15] mental health, [16–19] excess weight/obesity, [20–22] and infant mortality [23, 24].

Furthermore, research has shown that perceived discrimination, while seeking healthcare services, has robust links to chronic health conditions (e.g., heart disease, diabetes, and hypertension) and poor mental health outcomes (e.g., depression and psychiatric disorders). In particular, studies have suggested that perceived discrimination when seeking health care services is related to important care process factors such as health care utilization [2, 25, 26], communication between patient and provider [27, 28] and treatment adherence [29]. The Institute of Medicine’s report- Unequal Treatment: Confronting Racial and Ethnic Disparities in Healthcare - acknowledged that medical provider’s bias/prejudice is one mechanism for poor quality care and health outcomes among racial/ethnic minorities [30]. Yet, the study of discrimination in the healthcare setting is still in its infancy [5, 31]. The majority of the studies have examined the experiences of African Americans/Blacks and Hispanics [1]. There is a paucity of research on Asians as a whole or its specific groups [32], despite the fact that Asian Americans are one of the fastest growing populations in the United States [31].

Asian Indians (AIs) are the third largest Asian sub-group in the U.S., after Chinese and Filipinos, and one of the fastest growing ethnic minority group [33]. According to the 2010 Census, the U.S. is home to 3.2 million Asian Indians who are not confined to specific geographic areas in the US. In fact, Asian Indian were the largest detailed Asian subgroup in 23 states, more than any other detailed Asian group in 2010 [33]. Contrary to the model minority myth prevalent three decades earlier, Asian Indians have high prevalence rates of coronary heart disease (CHD), diabetes, and metabolic syndrome [34] and diverse linguistic, educational, religious and socio-economic characteristics [35, 36]. Despite their growing numbers and the reported high prevalence of chronic diseases, we are not aware of any research has explored perceived discrimination in medical care utilization in this high-risk ethnic group. In addition, prior studies on perceived discrimination among Asian immigrants have been narrow in scope or have aggregated multiple ethnic groups into the general category of “Asian Americans” [9, 37–39].

The lack of epidemiological data on health outcomes and perceived discrimination when seeking healthcare services among Asian Indians makes this study timely. Discrimination is a social stressor and physical health outcomes linked to discrimination may result in physiological responses such as elevated blood pressure and heart rate, which over time advance into chronic diseases such as CHD and hypertension [40].

The objectives of this study were to examine correlates of reported discrimination when seeking health care among a large sample of immigrant Asian Indian adults and to identify predictors of adverse self-rated physical health, a well-accepted measure of overall health status among individuals [41].

## Methods

### Participants and data collection

The data for this study were derived from the Diabetes among Indian Americans (DIA) study, the first national epidemiological survey of Asian Indians in the United States. A total of 1824 Asian Indians, aged 18 years and older, were interviewed from seven US cities with high concentration of Asian Indians - Houston, TX; Phoenix, AZ; Washington, DC; Boston, MA; San Diego, CA; Edison, NJ and Parsippany, NJ. The sampling procedure has been described in a prior publication [42]. Participation was voluntary, and informed consent was obtained from all subjects prior to participation. Telephone interviews were conducted by trained multilingual interviewers. The overall response rate was 37 % with 1824 Asian Indians completing the phone interview. Asian Indians that declined to participate in the study were requested to respond to a short questionnaire. Non-participants did not differ in gender, educational level, family history of diabetes and CVD or smoking status, but were significantly older than participants. The overall response rate was higher than in published health surveys of Asians or Asian Indians [43–46]. Additional information about the sampling frame and data collection for this study is available from a previous published study [34]. The study was approved by the institutional review board of Texas A&M University.

### Measures

#### Independent and outcome variables

Two primary variables of interest were assessed in our study, perceived discrimination when seeking health care and self-reported health status. Perceived discrimination when seeking health care, derived from questions from the Commonwealth Fund Health Quality Survey [47], was assessed by the following question: “Thinking of your experiences with receiving health care in the past 12 months, have you felt uncomfortable or been treated badly by your health care provider?” The responses were “Yes” “No” and “Unsure”. We created a binary variable where the “yes” responses were recoded as 1, and the “no” responses were recoded as 0. The “unsure” responses were excluded from the analyses because it does not unambiguously indicate the presence or absence of perceived discrimination; only 2 % (*n* = 32) of the sample indicated they were unsure. The second outcome in the study used the traditional self-rated health question: “Compared to others your age, how would you rate your overall physical health” with response options of “excellent, very good, good, fair, or poor” [48]. We created a binary variable by recoding “excellent/very good/good” as 0 and “fair/poor” as 1. Some of these questions have been used in publications from the DIA study [34, 42].

#### Covariates

Based on the existing literature, we included several covariates in our analyses [2, 5, 32, 49, 50]. Socio-demographic correlates included sex, age (18–34, 35–44, 45–54, ≥ 55 years), annual household income (< $24,999, $25,000–74,999, $75,000–$99,999, ≥$100,000), education (< high school graduate, some college or college graduate, graduate/professional), marital status (currently married, formerly married, never married). Additionally, we also included several health related correlates including health insurance (yes versus no), body mass index (Underweight/ normal: <24.9 kg/m^2^, Overweight: 25.00–29.9 kg//m^2^, Obese: ≥ 30 kg/m^2^), current cigarette and tobacco product use (yes versus no) and a count variable for sex-specific health conditions that included conditions of high blood cholesterol, cancer, diabetes, heart disease, high blood pressure, depression, arthritis, osteoporosis, kidney problems, thyroid problems, back problems (with a range of 0–9). Acculturation factors were included in the analysis since research shows acculturative stress can play a critical role in health among minorities; the two proxy acculturation measures were residency in the U.S. (<10 years vs. ≥ 10 years) and English language proficiency (speaks English very/pretty well versus not too well/not at all).

### Statistical analyses

After accounting for missing data across the variables used in the study, 892 respondents had no missing data for all of the study variables. As such, we further evaluated the item non-response for each of the variables with respect to the other variables. Compared to those with complete non-missing data (*n* = 892), independent t-test and chi-square analyses confirmed respondents with missing data (*n* = 932) did not significantly differ with regards to either outcome variable. However, there were some differences with respect to some of the covariates. Specifically, those respondents who were not missing on any of the variables were more likely (*p* < 0.05) to be male, live in a higher income household, have more education, speak English well, have health insurance and to be a current tobacco user when compared to the respondents who had a missing response on one or more of the variables. No differences (*p* > 0.05) however were noted between the two groups with respect to age, marital status, BMI or years lived in the US. Although we suspected that the data may have been missing at random, [51] we chose to impute data for missing cases using an iterative imputation method that imputed multiple variables by using chained equations, a sequence of univariate imputation methods with fully conditional specification of prediction equations [52, 53]. For the final post-imputation analyses, we used 10 imputed datasets of 1824 respondents in each datasets. All of the analyses in this study used STATA’s multiple (mi) estimation commands, which adjusted the coefficients and standard errors for the variability between the 10 imputed datasets according to the combination rules proposed by Rubin [54].

Respondent characteristics were summarized and are presented in Table 1. Following the analytic plan used in a similar study [2], we conducted bivariate and multivariable logistic regression analyses to examine the reports of perceived discrimination when seeking health care (Table 2) and poor self-rated health (Table 3). The bivariate analyses examined the relationship between perceived discrimination (Table 2) and each of the respondent’s characteristics separately. The multivariable logistic regression models assessed the adjusted odds ratios of reporting perceived discrimination (Table 2) and poor self-rated health (Table 3). All analyses were performed using the STATA software v13.1 (StataCorp LP, College Station, TX).

**Table 1:** Sample characteristics of analytic sample of American Indian (*n* = 1,824)

	N^b^	Percent
Perceived discrimination^a^		
No	1692	92.8 %
Yes	132	7.2 %
Self rated health		
Excellent/very good/good	1574	86.3 %
Fair/Poor	250	13.7 %
Sex		
Male	1103	60.5 %
Female	721	39.5 %
Age		
18–34	421	23.1 %
35–44	460	25.2 %
45–54	417	22.8 %
≥ 55	526	28.8 %
Annual household income		
< $25,000	229	12.6 %
$25,000–$74,999	550	30.2 %
$75,000–$99,999	389	21.3 %
≥ $100,000****	656	36.0 %
Education		
≤ High school graduate	172	9.4 %
College graduate	975	53.5 %
> College	676	37.1 %
Marital status		
Married	1632	89.5 %
Formerly married	83	4.6 %
Never married	109	6.0 %
Years lived in U.S.		
< 10 years	628	34.4 %
≥ 10 years	1196	65.6 %
English proficiency		
Very well/Pretty well	1635	89.6 %
Not too well/Not at all	189	10.4 %
Have health insurance		
No	261	14.3 %
Yes	1563	85.7 %
Body mass index^c^		
Underweight/normal	1015	55.6 %
Overweight	666	36.5 %
Obese	143	7.8 %
Current tobacco user		
No	1709	93.7 %
Yes	115	6.3 %
Chronic illness	Mean	SD
	1.03	0.03

Notes: ^a^Percieved discrimination when seeking healthcare services. ^b^N was calculated based on the estimated proportion. ^c^Underweight/normal (<24.9 kg/m^2^); Overweight (25.00–29.9 kg//m^2^) Obese (≥30 kg/m^2^)

**Table 2:** Correlates of poor treatment by health care provider among Indian Americans in the United States (*n* = 1,824)

	No. received poor treatment/No. in category (*n* ^a^ = 132/1,824)		Bivariate^b^ OR (95 % CI)		Adjusted^c^ OR (95 % CI)	
Self-rated health						
Excellent/very good/good	99/1,574	6.3 %	Reference		Reference	
Fair/Poor	33/250	13.3 %	2.29***	(1.48–3.54)	1.88*	(1.12–3.14)
Sex						
Male	73/1,103	6.6 %	Reference		Reference	
Female	59/721	8.2 %	1.26	(0.86–1.86)	1.36	(0.89–2.08)
Age						
18–34	31/421	7.4 %	Reference		Reference	
35–44	31/460	6.7 %	0.90	(0.52–1.59)	0.51****	(0.26–1.00)
45–54	26/417	6.1 %	0.82	(0.45–1.49)	0.32**	(0.15–0.69)
≥ 55	45/526	8.5 %	1.16	(0.66–2.03)	0.39*	(0.17–0.91)
Annual household income						
< $25,000	13/229	5.6 %	Reference		Reference	
$25,000–$74,999	51/550	9.3 %	1.76	(0.82–3.79)	1.33	(0.59–2.97)
$75,000–$99,999	27/389	6.8 %	1.25	(0.54–2.87)	0.90	(0.36–2.23)
≥ $100,000****	41/656	6.3 %	1.15	(0.54–2.46)	0.73	(0.31–1.69)
Education						
≤ High school graduate	6/172	3.5 %	Reference		Reference	
College graduate	82/975	8.4 %	2.63	(0.81–8.50)	2.44	(0.79–7.52)
> College	44/676	6.6 %	2.02	(0.64–6.41)	1.94	(0.62–6.02)
Marital status						
Married	118/1,632	7.3 %	Reference		Reference	
Formerly Married	6/83	6.9 %	0.89	(0.27–2.97)	0.66	(0.18–2.42)
Never Married	8/109	7.2 %	1.00	(0.46–2.14)	0.91	(0.38–2.14)
Have health insurance						
No	17/261	6.3 %	Reference		Reference	
Yes	116/1,563	7.4 %	1.19	(0.64–2.21)	0.88	(0.43–1.79)
Body mass index^d^						
Underweight/normal	70/1,015	6.9 %	Reference		Reference	
Overweight	47/666	7.1 %	1.03	(0.67–1.57)	0.93	(0.59–1.48)
Obese	15/143	10.3 %	1.52	(0.73–3.17)	0.94	(0.43–2.07)
Current tobacco user						
No	124/1,709	7.2 %	Reference		Reference	
Yes	8/115	7.3 %	1.01	(0.48–2.14)	0.97	(0.43–2.20)
Years lived in U.S.						
< 10 years	26/628	4.1 %	Reference		Reference	
≥ 10 years	106/1196	8.9 %	2.30**	(1.42–3.74)	3.28***	(1.73–6.22)
English proficiency						
Very/Pretty well	124/1,635	7.6 %	Reference		Reference	
Not too well/Not at all	8/189	4.1 %	2.07	(0.52–7.26)	1.61	(0.39–6.57)
Chronic illness	Mean	SD				
	1.6	1.5	1.38***	(1.20–1.57)	1.39***	(1.17–1.64)

*Abbreviations: No.* denotes number, *OR* denotes odds ratios, *CI* denotes confidence interval, *SD* denotes standard deviation

Notes: ^a^N was calculated based on the estimated proportion. ^b^Bivariate ORs reflect the association between Fair/Poor vs. good/very good/excellent and each of the covariates separately. ^c^Adjusted ORs reflect the association between Fair/Poor vs. good/very good/excellent, adjusting for all of the other variables. ^d^Underweight/normal (<24.9 kg/m^2^); Overweight (25.00–29.9 kg//m^2^) Obese (≥30 kg/m^2^). P-values: **** *p* < 0.10, *p < 0.05, **p < 0.01, ***p < 0.001

**Table 3:** Correlates of poor^a^ self-rated health among Indian Americans in the United States (*n* = 1,824)

	No. poor self-rated health^a^/No. in category (*n* ^b^ = 128/892)		Bivariate^c^ OR (95 % CI)		Adjusted^d^ OR (95 % CI)	
Perceived discrimination^a^						
No	109/714	12.8 %	Reference		Reference	
Yes	19/50	25.1 %	2.29***	(1.48–3.54)	1.93*	(1.17–3.19)
Sex						
Male	81/500	13.2 %	Reference		Reference	
Female	47/264	14.5 %	1.12	(0.84–1.49)	1.02	(0.73–1.42)
Age						
18–34	21/176	11.8 %	Reference		Reference	
35–44	35/215	11.9 %	1.01	(0.65–1.57)	0.71	(0.43–1.18)
45–54	34/163	17.2 %	1.56^+^	(1.00–2.43)	0.84	(0.47–1.49)
≥ 55	38/210	14.0 %	1.22	(0.80–1.87)	0.48*	(0.26–0.89)
Annual household income						
< $25,000	21/60	20.9 %	Reference		Reference	
$25,000–$74,999	28/224	11.9 %	0.51*	(0.31–0.85)	0.56*	(0.32–1.00)
$75,000–$99,999	27/178	12.7 %	0.55*	(0.33–0.93)	0.74	(0.37–1.47)
≥ $100,000^+^	52/302	13.2 %	0.58*	(0.37–0.90)	0.74	(0.38–1.43)
Education						
≤ High school graduate	16/41	18.9 %	Reference		Reference	
College graduate	72/402	13.8 %	0.69	(0.43–1.09)	0.93	(0.52–1.67)
> College	40/321	12.2 %	0.60*	(0.37–0.95)	0.84	(0.45–1.57)
Marital status						
Married	27/76	14.1 %	Reference		Reference	
Formerly Married	101/688	12.3 %	0.84	(0.36–1.93)	0.65	(0.26–1.59)
Never Married		8.0 %	0.52	(0.25–1.13)	0.59	(0.25–1.38)
Have health insurance	121/691					
No	3/28	20.8 %	Reference		Reference	
Yes	4/45	12.5 %	0.54**	(0.37–0.80)	0.56*	(0.35–0.92)
Body mass index^e^						
Underweight/normal	56/446	11.0 %	Reference		Reference	
Overweight	54/275	14.6 %	1.39*	(1.01–1.90)	1.23	(0.88–1.71)
Obese	18/43	28.0 %	3.14***	(2.03–4.88)	2.13**	(1.32–3.42)
Current tobacco user						
No	114/721	13.1 %	Reference		Reference	
Yes	14/43	22.7 %	1.95**	(1.23–3.10)	1.80*	(1.09–2.98)
Years lived in U.S.						
< 10 years	32/253	11.9 %	Reference		Reference	
≥ 10 years	96/511	14.6 %	1.27	(0.93–1.72)	1.35	(0.86–2.12)
English proficiency						
Very/Pretty well	213/1,635	13.0 %	Reference		Reference	
Not too well/Not at all	37/189	19.4 %	0.62*	(0.41–0.96)	0.93	(0.48–1.80)
Chronic illness	Mean	SD				
Received poor treatment	1.6	1.5	1.44***	(1.30–1.60)	1.46***	(1.28–1.66)

*Abbreviations: No.* denotes number, *OR* denotes odds ratios, *CI* denotes confidence interval, *SD* denotes standard deviation

Notes: ^a^Includes poor/fair responses. ^b^N was calculated based on the estimated proportion. ^c^Bivariate ORs reflect the association between Fair/Poor vs. good/very good/excellent and each of the covariates separately. ^d^Adjusted ORs reflect the association between Fair/Poor vs. good/very good/excellent, adjusting for all of the other variables. ^e^Underweight/normal (<24.9 kg/m^2^); Overweight (25.00–29.9 kg//m^2^) Obese (≥30 kg/m^2^). P-values: ^+^ *p* < 0.10, *p < 0.05, **p < 0.01, ***p < 0.001

## Results

The sample was comprised of Asian Indian men and women between 18 and 88 years of old (*n* = 1824). The sample characteristics are presented in Table 1. A small proportion of participants (approximately 7 %) reported perceived discrimination when seeking health care and 14 % reported poor (fair or poor) self-rated health. The majority of participants were males (60 %), 45 years of age and older (50 %), (higher socioeconomic status with reported annual household income of more than $77,000 (~50 %), had a college degree or a higher (90 %), and were married (90 %).

In terms of acculturation status, approximately two-thirds of the sample lived in the US for more than 10 years and 90 % reported speaking English very well or pretty well. Given the relatively high socioeconomic status, it is not surprising that approximately 85 % of the sample reported having health insurance. The majority of the sample reported no tobacco use (93.7 %) and, on average, had only one chronic illness (1.03 ± 0.03). Although the prevalence of obesity was not as high as the general U.S. population, almost one in every three respondents were overweight based on their BMI.

Results from the bivariate and multivariable logistic regression analyses are presented in Table 2. Although perceived discrimination when seeking health care were reported by a relatively small proportion of the population (7.2 %), the patterns of report was very instructive with respect to some of the covariates. For example, reports of discrimination were associated with age, self-rated health, acculturation and presence of chronic illnesses. The results between the unadjusted and adjusted ORs were consistent, with the exception of age; age was statistically significant in the adjusted models but not in the unadjusted models. As shown in the adjusted model, respondents who reported fair/poor health were more likely to report experiencing discrimination when seeking healthcare services. Specifically, respondents who reported fair/poor self-rated health were approximately twice as likely to perceived discrimination when seeking care as compared to those in good or excellent health status (OR 1.88; 95 % CI 1.12–3.14). Furthermore, Asian Indians who lived for more than 10 years in the U.S. (OR 3.28; 95 % CI: 1.73–6.22) and had chronic illnesses (OR 1.39; 95 % CI: 1.17–1.64) (*p* < 0.05) were more likely to perceive discrimination when seeking health care. However, older Asian Indians were less likely to perceive discrimination than those aged 18–34 years.

The relationships of self-rated health status, perceived discrimination when seeking health care and the respondent characteristics are presented in Table 3. Poor self-rated health was associated with perceived health care discrimination after controlling for all of the respondent characteristics in the model (OR 1.93; 95 % CI: 1.17–3.19). Additional respondent characteristics that were positively associated with poor self-rated health included younger age (18–34 years old compared to >54 years old), low household income (< $25,000 compared to $25,000–$74,999), having no health insurance, being obese, current use of tobacco products, and having one or more chronic illness. Much like the results from the analyses of perceived discrimination when seeking health care (Table 2), the odds ratios between unadjusted and adjusted analysis remained relatively consistent in magnitude with the exception of age. In the unadjusted model, although the coefficients were not statistically significant (*p* < 0.05), older Asian Indians were more likely to report poor health as compared to those aged 18–34 years. However, after adjusting for all the demographic characteristics, the coefficients reversed directions. In particular, older Asian Indians, over the age of 55 years, were less likely (OR 0.48; 95 % CI: 0.26–0.89) to report being in poor health than their younger counterparts (18–34 years old).

## Discussion

The results of this study contributes to a growing body of evidence which suggests that Asian Indians, similar to other racial/ethnic minority groups in the U.S., experience discrimination while seeking health care services [5, 15]. Although the reports of perceived discrimination when seeking health care services were relatively low (7.2 %) in this population, compared to other racial/ethnic minorities in the US, [1] it is similar to findings by Southeast Asians (7.5 %) and Pacific Islanders (3.0–9.1 %) as reported in previous studies [25, 26, 55–57]. In our sample, reports of health care discrimination ranged from 3.5 % among those with less than a college degree to 13.3 % among those with fair/poor self-rated health status. Similar to other studies, results from the multivariable analyses suggest that Asian Indians who reported fair or poor health were more likely to report discrimination when seeking health care services, adjusting for various confounders in the model [2, 26, 58].

Results also suggested that personal characteristics such as age, length of residency in the US and chronic illness were all predictors of perceived discrimination. For example, age was inversely associated with reports of discrimination while seeking health care [2], which is relatively consistent with other studies of non-healthcare discrimination [5]. Prior studies suggest that older individuals who grew up in the oppressive civil rights era are less likely to perceived discrimination in today’s society in light of the current civil rights enjoyed by racial/ethnic minorities today [32, 49 50]. An alternate explanation is that older Asian Indians may have been exposed to greater unfair events and have developed adequate coping skills to deal with the effects of discrimination. Having coping skills appears to reduce the effects of discrimination and resiliance among minorities [59]. This cultural resilience is often fostered by protective factors of new experiences, opportunities and cultural connectedness with ethnic community networks to neutralize or offset the detrimental effect of discrimination to reduce stress [60]. Hence, cultural resilience has been regarded as a potentially positive resource for compensating the detrimental effect of stress and social risk factors to reduce discrimination and improve social outcomes. Furthermore, cultural resiliance is positively associated with better mental health status even among forced migrants [61]. It is also possible that younger Asian Indians, who tend to be new immigrants, may be more susceptible to discrimination sensitization due to a greater psychological distress for acculturation to the American culture, occupational issues, or group identification. Acculturation, a complex phenomeon, is defined as the extent to which immigrants adapt to the host culture in comparison to retaining their ethnic culture [62]. The degree of acculturation and time spent in the United States among immigrant AIs can play a significant factor in the psychological distress [63] and are associated with self-reported experiences of racial discrimination especially among acculturated individuals [63]. While health of foreign-born individuals are reported to be better than native-borns in the US [64], the healthy immigrant effect slowly wanes with convergence of health to native-borns with length of residency; structural and contextual factors such as social and economic inequalities also influence the perception of discrimination and shape health status [65]. New immigrants are less likely to interact with the health systems of their host countries then native-borns but with increasing length of stay there is a narrowing the utilization of health services [64].

Past research has revealed that social support, coping style, and ethnic identity moderate the link between perceived discrimination and health [5]. If indeed, younger Asian Indians percieved greater discrimination in the health care system, this is particularly troubling if such perceptions eventually leads to the under-ulilization of health sercives. Studies on Southeast Asians in the United States have found that forbearance or emotion-focused coping diminished discrimination-related depression [19]. However, efficacy of emotion-focused coping for Asian Indians my be lessened when younger individuals adapt to the Western environment [66]. Similarly, Asian Indians who have lived in the United States for more than 10 years were more than three times as likely to percieved health care discrimination when compared to those who were in the US for less than 10 years, supporting the notion that discrimination may be positively associated with length of time lived in the US among other Asian subgroups in the US [67]. Future research should focus on the potential consequences of percieved discrimination in the health care settings, such as adherence and untilization.

Our results also highlighted that experiences of health care discrimination are associated with fair or poor self-rated health status. Asian Indians who perceived discrimination were twice as likely (OR 1.93; 95 % CI: 1.17–3.19) to report fair or poor health status as compared to those that did not report any discrimination, controlling for important demographic characteristics. Although Asian Indian women were not significantly more likely to report fair or poor self-rated health, prior studies indicated Asian American women reported slightly higher rates of fair to poor-self rated health status than men [68]. Perceived health care discrimination increased the odds of chronic illness and corroborates with population-based health studies [31, 38, 69]. Stronger associations are consistently found for mental health than physical health outcomes suggesting a higher discrimination threshold may be needed for physical health effects [70].

The findings of the present study should be considered in the context of several limitations. First, this study used cross-sectional data; therefore, our understanding of the causal relationship we examined is limited. Second, the data used are from select large cities in the US. Although the study employed a sampling procedure to recruit a large sample of Asian Indians, future research is needed to determine whether our findings are generalizable to the larger population of Asian Indians in the US. Finally, we were not able to ascertain the perceived reasons for the poor treatment (e.g. race/ethnicity, age, etc.) due to the relatively small proportion indicating that they were treated unfairly. Hence, further research is necessary to examine this particular question among this fastest growing subgroup of Asians in the U.S.

## Conclusions

In conclusion, this study offers initial support for the hypothesis that Asian Indians experience discrimination when seeking health care services and that these experiences may be related to poor self-rated health status. Although Asians in general and Asian Indians in particular have higher levels of education and income, they may not be perceived as having vulnerability to the experiences of discrimination in healthcare settings. As noted, additional studies that explore the role of perceived interpersonal discrimination in medical care utilization among Asian Indians in the US is warranted. This particular line of inquiry should continue to be of interest because Asian Indians are the third largest sub-group of Asians in the US. Indeed, if perceived interpersonal discrimination when seeking healthcare services impacts future healthcare utilization, this may exacerbate the overall health burden of the Asian Indian population in the US given that chronic health conditions are relatively high among this ethnic group despite their relatively high socioeconomic status. This study also highlights the need to include Asian Indians in future research that examines the impact of health care utilization policies, like cultural competency training among healthcare employees.

Additionally, future research should also examine the dimension and type of discrimination as well as protective factors that can moderate between discrimination and physical and mental health outcomes. This will refine our knowledge base and guide policies and strategies, while acknowledging the heterogeneity within Asians and Asian Indians in order to reduce health disparities in this fastest growing subgroup of Asians in the U.S.
